# Proton range verification with MACACO II Compton camera enhanced by a neural network for event selection

**DOI:** 10.1038/s41598-021-88812-5

**Published:** 2021-04-29

**Authors:** Enrique Muñoz, Ana Ros, Marina Borja-Lloret, John Barrio, Peter Dendooven, Josep F. Oliver, Ikechi Ozoemelam, Jorge Roser, Gabriela Llosá

**Affiliations:** 1 Instituto de Física Corpuscular (IFIC), CSIC/Universitat de València, València, Spain; 2 KVI-Center for Advanced Radiation Technology, University of Groningen, Groningen, The Netherlands; 3 Department of Radiation Oncology, University Medical Center Groningen, University of Groningen, Groningen, Netherlands

**Keywords:** Imaging techniques, Experimental particle physics, Radiotherapy

## Abstract

The applicability extent of hadron therapy for tumor treatment is currently limited by the lack of reliable online monitoring techniques. An active topic of investigation is the research of monitoring systems based on the detection of secondary radiation produced during treatment. MACACO, a multi-layer Compton camera based on LaBr_3_ scintillator crystals and SiPMs, is being developed at IFIC-Valencia for this purpose. This work reports the results obtained from measurements of a 150 MeV proton beam impinging on a PMMA target. A neural network trained on Monte Carlo simulations is used for event selection, increasing the signal to background ratio before image reconstruction. Images of the measured prompt gamma distributions are reconstructed by means of a spectral reconstruction code, through which the 4.439 MeV spectral line is resolved. Images of the emission distribution at this energy are reconstructed, allowing calculation of the distal fall-off and identification of target displacements of 3 mm.

## Introduction

Hadron therapy is a growing technique that has been employed to treat over 350,000 cancer patients worldwide^1^. The reason for its increasing importance is its beneficial dose deposition profile, which enables highly focalised treatments and better sparing of the surrounding healthy tissue. However, the high concentration of the delivered dose at the Bragg peak makes hadron therapy very sensitive to any source of deviation from the treatment planning or to uncertainties in the plan itself. As a consequence, additional safety margins of several millimeters are set in the treatment planning in order to ensure that the clinical volume target receives the prescribed dose and that organs at risk are not irradiated^2^. The reduction of safety margins would allow a better delineation of the irradiated volume and a wider use of hadron therapy in tumors close to organs at risk. This can be achieved by monitoring online the particle range inside the patient.

Online monitoring can be realized by detecting the secondary radiation emitted along the beam path. Secondary particles are produced in the de-excitation of nuclei and nuclear fragments formed in the interactions between the beam and the irradiated tissue. Positron Emission Tomography (PET) has already been used clinically to monitor the distributions of $\beta$+ emitters generated during irradiation^3–5^. However, PET imaging presents some intrinsic drawbacks: displacement of $\beta$+ emitters due to biological washout, delayed signal with respect to irradiation time due to the half-lives of the most abundant $\beta$+ emitters and the high background of prompt emissions^6,7^. In addition, PET imaging devices must be adapted for in-beam imaging, requiring a dedicated design to integrate the scanner with the beam^8^. To overcome the delayed emission and displacement caused by washout, PET imaging of very short-lived $\beta$+ emitters is currently under investigation^9–11^. Alternatively, the prompt emission of high energy photons or prompt gamma-rays (PG), which do not suffer from the mentioned drawbacks, can also potentially be used for online verification^12,13^.

The PG emission spectrum exhibits a decreasing continuum, extending up to several MeV, with the presence of prominent lines corresponding to discrete transitions between nuclear energy levels^14^. In particular, an intense peak of emission is found at 4.439 MeV, originating from the de-excitation of $^{12}$C to its ground state. During irradiation, the excited state of $^{12}$C is produced following inelastic proton scattering off $^{12}$C and proton induced spallation of $^{16}$O^15^, both of which are relatively abundant in human tissues. The cross section of the involved nuclear reactions have a maximum at low proton energies^16^. As a consequence, the emission distribution of PG at this energy presents a peak of intensity near the end of the beam range and can be correlated to the delivered dose.

Several monitoring techniques based on the detection of PG are currently under investigation. Mechanical collimation has been proposed with different collimator geometries^17–20^. Clinical tests with a collimated gamma camera have also been reported^21^. However, a disadvantage of this modality is the collimator thickness required to fully absorb the high energy PG, which reduces drastically the detection efficiency. Other methods based on measuring the PG time of flight distribution, correlated to the ion range inside the patient, are also being investigated^22,23^. A review of the state of the art in hadron therapy monitoring through PG can be found in^24^. Another imaging modality currently under study for treatment monitoring is based on electronic collimation. Compton imagers have emerged as a promising candidate due to their ability to image photons at the PG energy range and the fact that they do not require mechanical collimation. Compton cameras are also being investigated for Single Photon Emission Computed Tomography (SPECT) with high efficiency^25–27^. In Compton imaging, the initial energy of a measured photon must be known in order to constrain its origin to a conical surface containing its emission position. To deal with the polychromatic nature of the PG spectrum, Compton imaging can be achieved by detecting three-interaction events, which allow reconstruction of the initial energy photon energy^28,29^. If only two-interaction events are detected, images can be reconstructed by selecting well-known PG emission lines, requiring full energy absorption in the second interaction or employing spectral reconstruction methods to estimate both the spatial and the spectral emission distribution^30–32^. Different Compton camera prototypes and reconstruction methods have been designed for this purpose^33–38^. To the end of reconstructing the measured activity distribution by means of a Compton camera, the background component in the measured data must be taken into account. Event selection for background reduction is usually employed in Compton imaging, both in its application to astronomy^39^ and to medical imaging^40^. Event selection is of great importance in PG reconstruction for hadron therapy monitoring, since high intensity clinical beams can lead to an increase of random coincidences produced by different incoming particles^41^. In addition to photons, a high background of neutrons and protons can reach the detectors, triggering the acquisition of unwanted events that contaminate the true signal.

At IFIC-Valencia, the MACACO (Medical Applications CompAct COmpton camera) prototype is being developed for the purpose of hadron therapy online treatment monitoring. It is a three-plane Compton camera based on LaBr$_3$ crystals coupled to silicon photo-multiplier (SiPM) arrays^42^. The first prototype was previously fully characterised and tested in the laboratory with a variety of sources^43^ and in-beam^44,45^. The promising results obtained with the first version led to the development of MACACO II, a second prototype with enhanced energy resolution^46^. This paper presents the results obtained in-beam with MACACO II operated with two detector planes and using a spectral method to estimate the PG energy. The experiment was conducted at the irradiation facility of the AGOR cyclotron at KVI-Center for Advanced Radiation Technology, University of Groningen. A Polymethyl methacrylate (PMMA) target placed at different locations with respect to the camera was irradiated by a 150 MeV proton beam.

In this paper, a fully connected neural network (NN) is employed for event selection. NNs have been previously applied to identification of sequence ordering in a multi-layer Compton telescope^47^. In this work, the implemented NN is trained to discriminate signal from background events, and a simulation study is performed to demonstrate that this discrimination improves identification of the PG peak of emission. Finally, the trained NN is used for background reduction in the experimental data. Images are reconstructed using a spectral reconstruction method for two-plane Compton cameras^32^, with particular emphasis on the 4.439 MeV PG emission line. The reconstructed images and profiles are presented, demonstrating the system capability of detecting range shifts of 3 mm.

## Results

### Event selection with simulated data employing a neural network

The event selection procedure is performed by means of a NN, described in the event selection subsection of the methods section. A total of $1.2 \cdot 10^6$ events were obtained from Monte Carlo simulations using GATE^48^, labeled as true signal or background events as explained in the simulations subsection. The simulated dataset was divided into two equally sized training and validation sets with $6.1 \cdot 10^5$ events each. The NN parameters are optimized with the training dataset, and the validation dataset is then used to analyse the NN performance. The percentage of true signal and background events in the validation set before and after event selection by the trained NN is shown in Table 1.

**Table 1:** Signal and background events in the validation set before and after NN selection.

	Total	True signal	Background	Percentage of true signal (%)
Events before selection	$6.1 \cdot 10^5$	$4.7 \cdot 10^4$	$5.6 \cdot 10^5$	7.6
NN selected events	$3.8 \cdot 10^5$	$3.9 \cdot 10^4$	$3.4 \cdot 10^5$	10.3

**Figure 1:**
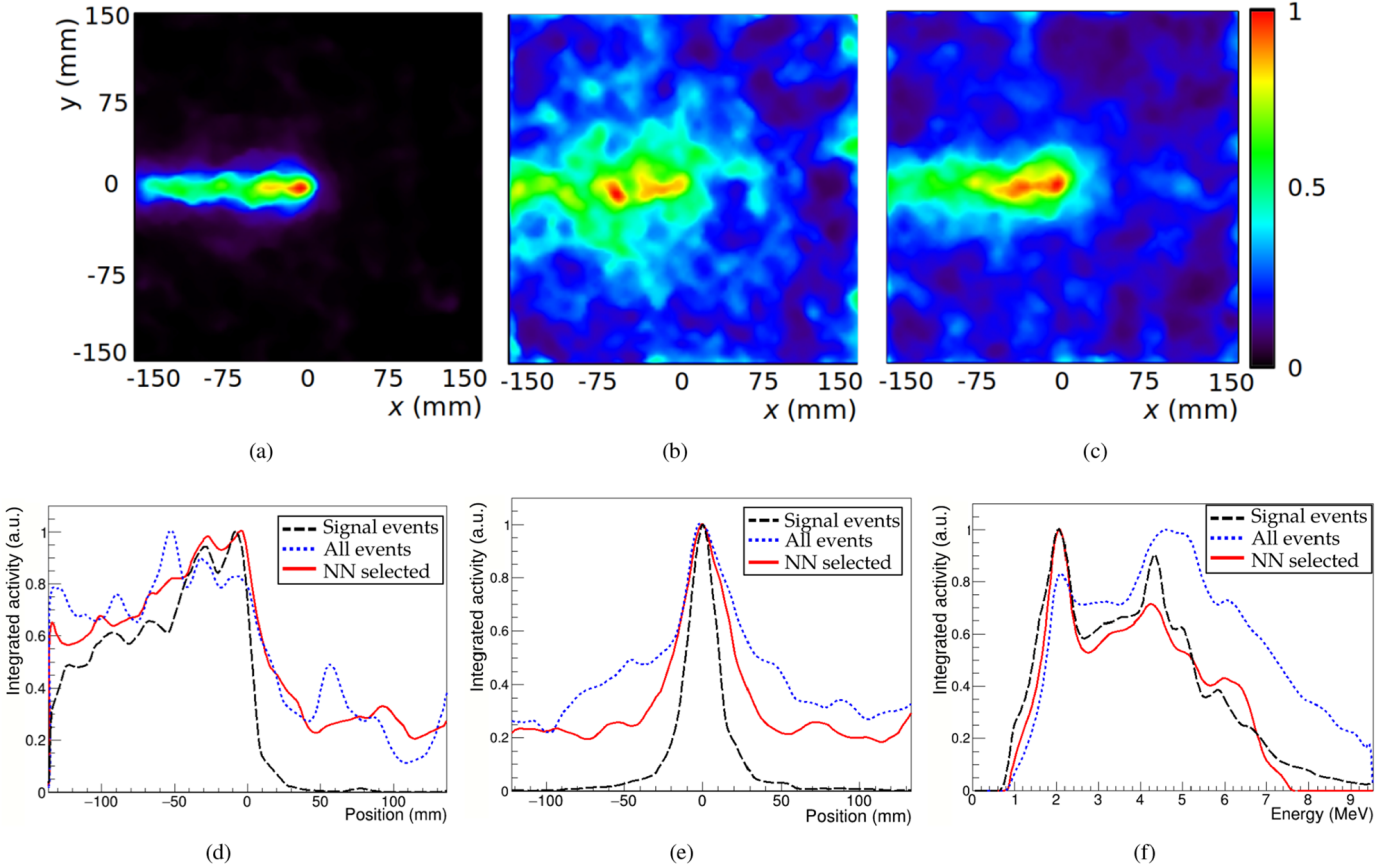
Reconstructed images at 4.4 MeV using three different simulated data sets: only true signal events (**a**), all events (**b**) and events selected by the trained NN (**c**). (**d**) Line profiles along the beam direction from the above images. (**e**) Transverse line profiles at the maximum position. (**f**) Energy spectra recovered after integration over the reconstructed image spatial domain.

Events in the validation set are selected by the trained NN with a recall (percentage of the total true signal correctly classified) of 84.0% and a precision (percentage of true signal over the accepted events) of 10.3%, improving the percentage of signal from the original 7.6% registered in the raw data. This constitutes a relative increase of 35.4% in the ratio of signal events, which has an important effect in the reconstructed images.

**Table 2:** Studied parameters of the reconstructed images with the three simulated datasets.

	Signal events	All events	NN selected
Relative Max, R80 and R50 positions (mm)	–	–44.8 $\vert$ –41.3 $\vert$ 13.1	3.0 $\vert$ 4.1 $\vert$ 11.1
Relative value at true peak position	1	0.83	0.98
Relative average background value	(6.3 ± 9.6) $\cdot 10^{-3}$	0.24 ± 0.15	0.18 ± 0.10
Contrast-to-noise ratio	100	4.0	7.7
FWHM in transverse beam direction (mm)	19.6 ± 0.8	63 ± 3	47 ± 2
Reconstructed energy peak (MeV)	4.4 ± 0.4	4.8 ± 1.2	4.3 ± 0.7

Images of the photon emission distribution produced by the simulated proton irradiation have been reconstructed using three different sets of data extracted from the validation set: only true signal, all events and events selected by the NN. The images obtained at the energy of 4.4 MeV are shown in Figs. 1a,b,c. The longitudinal profiles along the beam direction are plotted in Fig. 1d, where the reconstructed emission peaks can be identified. Different parameters have been calculated from the reconstructed images in order to assess the effect of the event selection technique (see the image assessment and range estimation subsection). The calculated parameters are listed in Table 2. All the parameters obtained with NN selected events improve with respect to those obtained with all events. The peak height and position along the beam direction are calculated relative to the values obtained using only signal events. As seen from Fig. 1d, the maximum peak position using all events is displaced by 45 mm, while the deviation obtained from the selected events is reduced to 3 mm. In the transverse beam direction, the full with at half maximum (FWHM) is also reduced with this dataset, showing a better reconstruction of the beam lateral spread. This can be seen in Fig. 1e, where the transverse profiles from the three datasets are plotted. The image reconstructed with selected events also assigns a higher value at the true peak position, and the background voxels outside the region of interest have lower average value and standard deviation. This results in an enhanced contrast-to-noise ratio (CNR), improving from 4.0 to 7.7. Finally, the reconstructed energy spectra in the three cases are also plotted in Fig. 1f. In this plot, a better identification of the 4.4 MeV line is also obtained with the NN selected events. Gaussian fits to the peaks around this energy yield a mean and sigma of $4.3 \pm 0.7$ MeV when NN selected events are used, improving from the $4.8 \pm 1.2$ MeV calculated with all events. The $R^2$ coefficients of determination of these fits range from 0.9798 to 0.9947.

### Reconstructed images with experimental data

Images of the distribution of photons generated by the proton beam irradiation of a PMMA target placed at different positions with respect to the prototype have been reconstructed. An initial measurement was performed with high statistics in time coincidence to obtain a detailed reconstruction of the PG emission distribution profile. In addition, six more measurements at different positions were performed aimed at the identification of shifts in the Bragg peak location. In order to emulate shifts in the Bragg peak without altering the beam parameters, measurements were taken with the target at different positions with respect to the system. Data were taken with target displacements of ±2, ±5 and ±25 mm along the beam direction, with a precision of ±5 $\mu$m. The measurement time was 2 hours for the initial measurement and 35 minutes for each of the six measurements at different positions.

**Figure 2:**
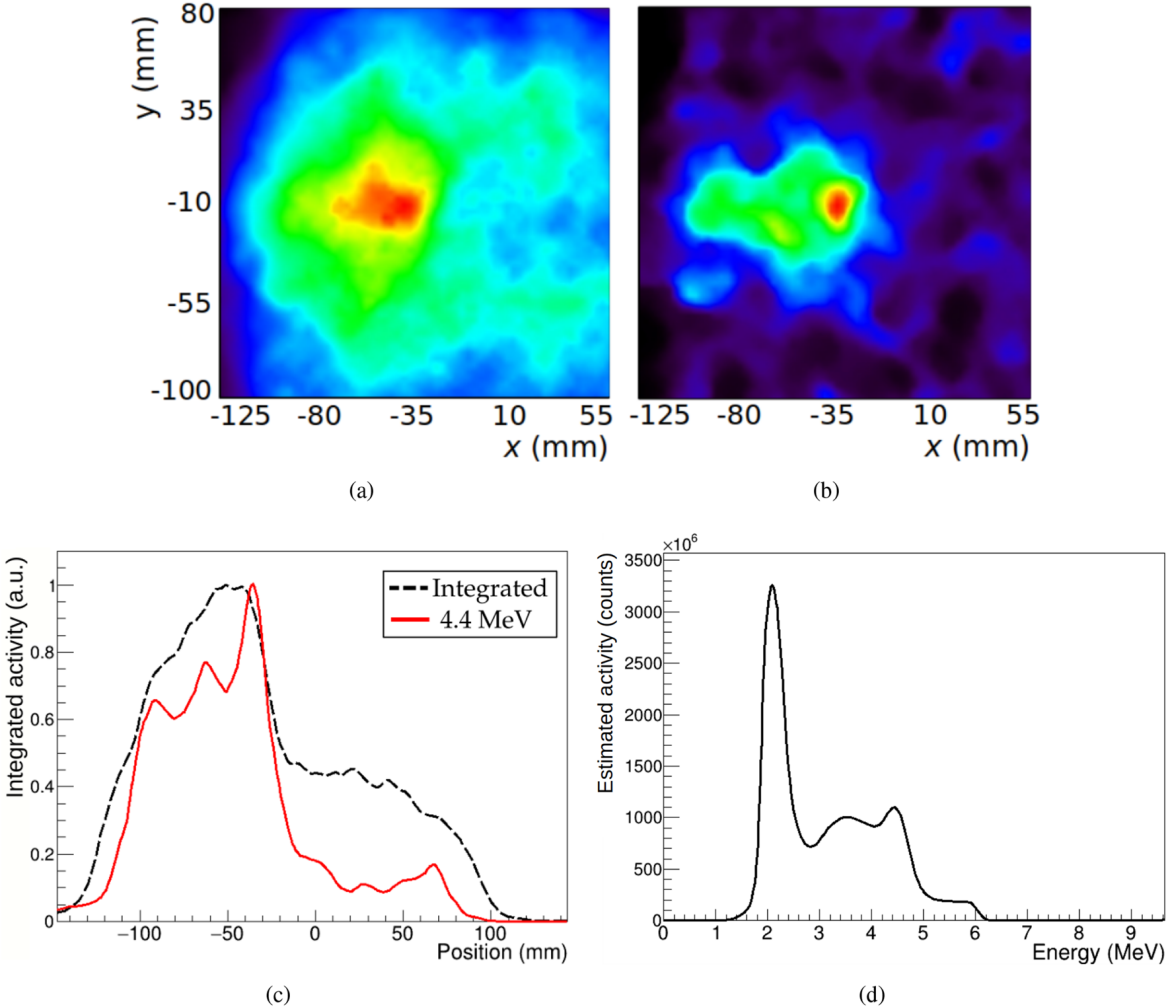
Reconstructed image from measurement at position 0. (**a**) Image integrated over the whole spectral domain. (**b**) Image obtained at the energy slices of 4.4 MeV. (**c**) Line profiles along the beam direction from the above images: whole energy range (black dashed line) and 4.4 MeV (red solid line). (**d**) Energy Spectrum recovered after integration over the reconstructed image spatial domain.

The NN trained in the previous simulation study was also employed for event selection in the experimentally measured data. The number of events used for image reconstruction, after selection with the NN, was $13.9 \cdot 10^4$ events for the 0 mm position and $(4.11 \pm 0.07) \cdot 10^4$ on average for the six measurements with target displacements.

Images reconstructed from the measurement with higher statistics (position 0) are shown in Fig. 2. Figure 2a,b show the spatial distributions obtained after integration over the whole spectral domain and at 4.4 MeV, calculated by integrating the reconstructed image between 4.3-4.5 MeV. The reconstructed spectrum is plotted in Fig. 2d. This spectrum is obtained after integration of the spatial domain in the reconstructed images, and represents the emission activity of PG estimated from the data employed for reconstruction. As in the reconstructed spatial images, this estimation is contaminated by the background present in the measured dataset. Nevertheless, a local maximum can be appreciated at 4.4 MeV. The peak at 2.2 MeV corresponds to the line produced by the de-excitation of deuterium after neutron capture by hydrogen. Since this distribution is not correlated to the primary proton range^24^, it is not expected to contain usable beam information, and it has not been employed in this work for the determination of target shifts. The 2.2 MeV line is the main contribution to the high background in the image integrated over the whole energy range. A region of higher activity at the expected peak position can be seen in this image, but the wide spread of intensity over the whole image makes it difficult to estimate the beam range from it. The beam direction and range are much better defined in the 4.4 MeV image, where the emission intensity increases steadily until reaching a peak, after which the intensity quickly drops. This can be better appreciated in the longitudinal profiles plotted in Fig. 2c.

**Figure 3:**
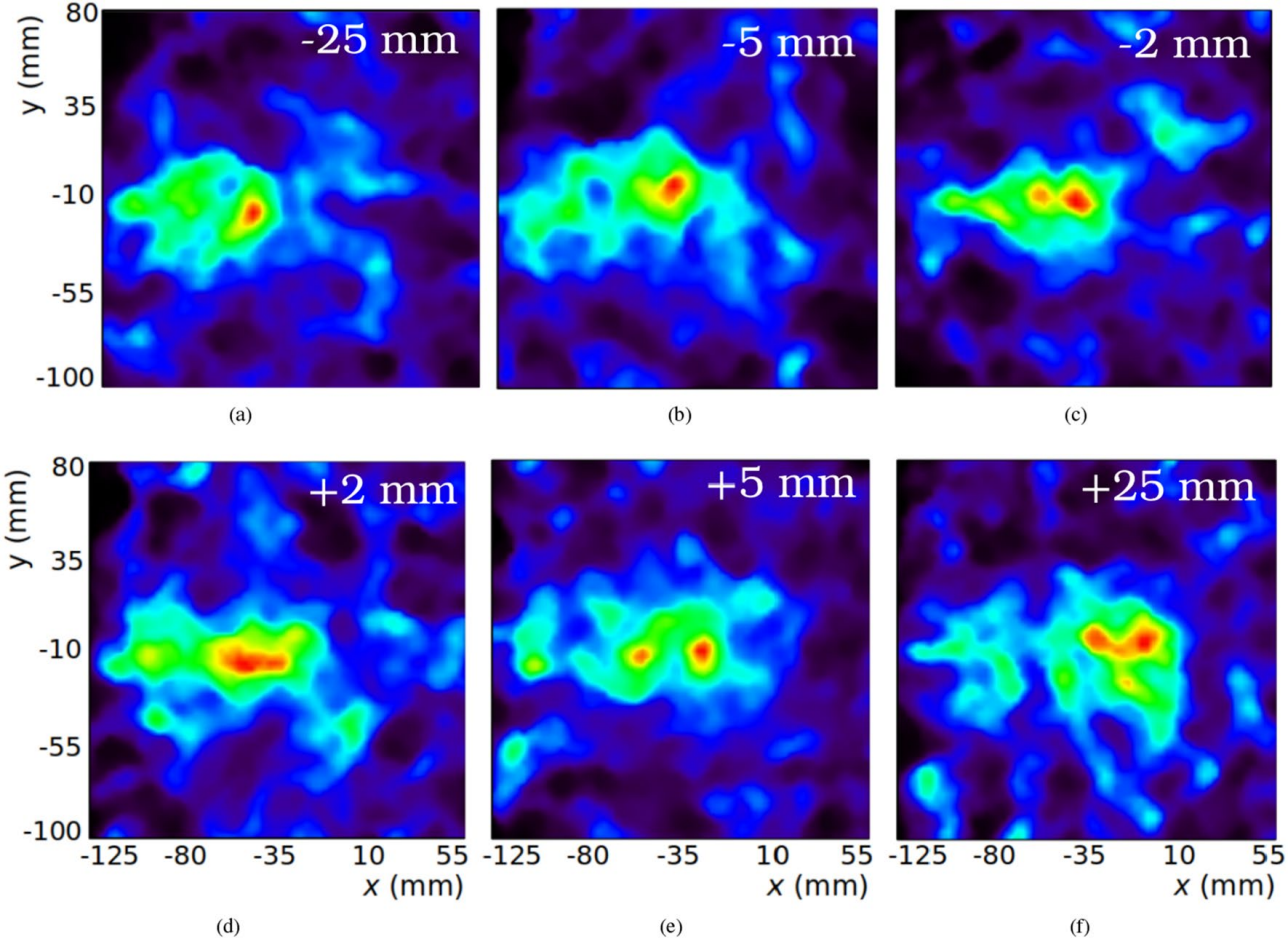
Reconstructed images at 4.4 MeV at 6 different target positions. Top row, from left to right: target at –25 (**a**), –5 (**b**) and –2 mm (**c**). Bottom row, from left to right: target at +2 (**d**), +5 (**e**) and +25 mm (**f**).

The reconstructed spatial images corresponding to target displacements at the energy of 4.4 MeV are shown in Fig. 3. Since these measurements have lower statistics than the previous one, the images obtained are noisier, leading to local peaks in the reconstruction. Nevertheless, the emission distribution along the beam direction, as well as a peak of intensity at the end of the beam range, can be seen in all cases. The calculated lateral spread of the reconstructed beam in these figures is 46 ± 3 mm FWHM on average, degraded from the 35 ± 1 mm obtained from the reference image with higher statistics. These images also present a higher background level, causing the CNR to be degraded from 6.9 in the reference image to 5.7 ± 0.3 for the six displaced positions. Regarding the reconstructed emission spectra, the reconstructed peak position was 4.5 ± 0.4 MeV on average, demonstrating that the PG emission line at 4.4 MeV can also be recovered from these measurements.

**Figure 4:**
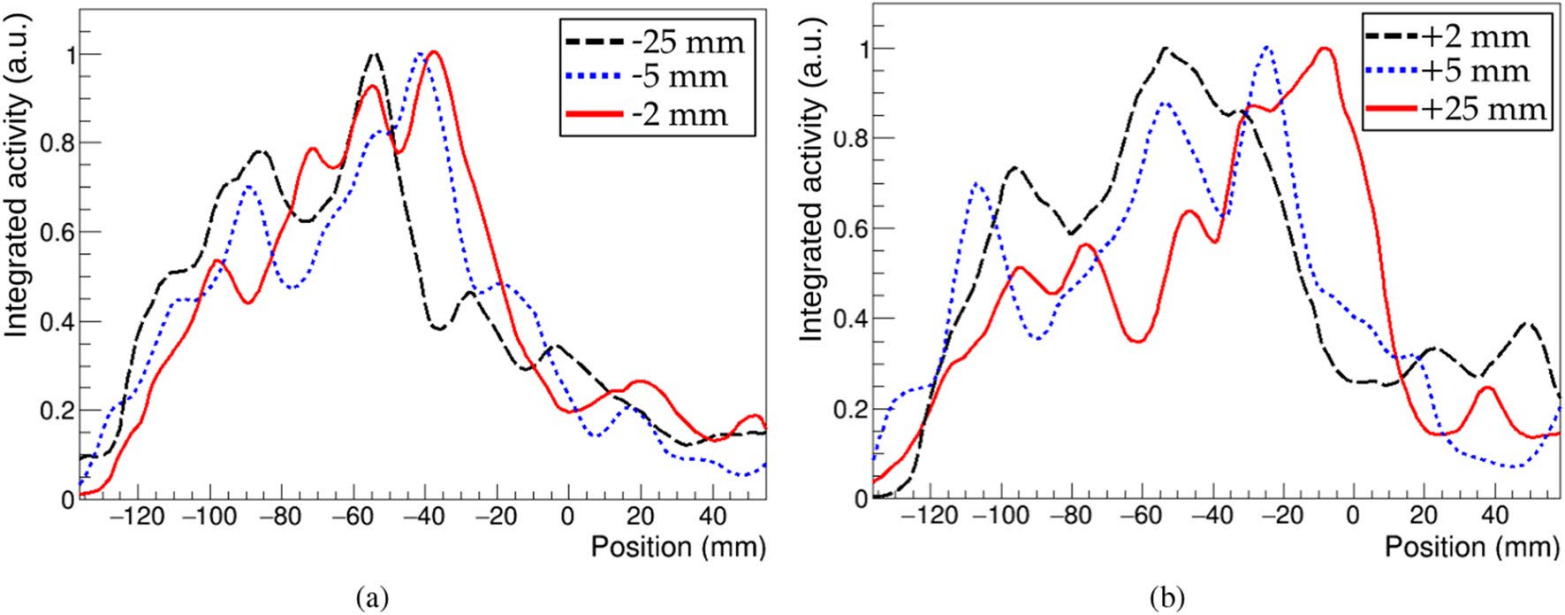
Line profiles along the beam direction from images at 4.4 MeV shown in Fig. 3: (**a**) top row, (**b**) bottom row.

In order to estimate the range from the different images, the position of the maximum of the reconstructed photon emission distribution and the positions at R80 and R50 after the maximum are calculated. These features are obtained from the longitudinal profiles along the beam direction, plotted in Fig. 4. Figure 4a,b plot together the longitudinal profiles obtained from measurements in which the PMMA target was shifted to the left (–25, –5 and –2 mm) and right (+2, +5 and +25 mm) of the central position, respectively. From all profiles, it can be seen that the distal edge is shifted in agreement with the displacement of the target along the beam direction. Relative displacements at the different target positions have been quantified taking as a reference the values obtained for each of the three parameters (maximum, R80 and R50) at position 0. These values at position 0 are –35.7 mm for the maximum peak, –29.9 mm for R80 and –23.4 mm for R50. Considering these relative displacements, the best results are achieved using the R80 parameter, with an average relative deviation from the expected values of 3.4 mm (2.2 mm if we consider only the central positions, with target displacements between –5 and 5 mm). The average relative deviations from the expected values obtained using the other two parameters is 6.2 mm for the maximum peak and 5.5 mm for R50. All the parameters calculated for each of the images are listed in Table 3.

**Table 3:** Calculated parameters from the images reconstructed with measurements at different target positions. Positions along beam direction are calculated relative to the values obtained at position 0. Average values on the last row are calculated excluding position 0.

Target position (mm)	Relative position (mm)			Lateral spread	CNR	Energy peak (MeV)
	Max	R80	R50	FWHM (mm)		
0	–	–	–	35 ± 1	6.9	4.4 ± 0.3
–25	–17.9	–18.7	–18.1	42 ± 1	6.8	4.4 ± 0.3
–5	–5.7	–4.7	–4.0	47 ± 1	5.8	4.4 ± 0.4
–2	–3.0	–0.2	+4.0	38 ± 3	5.5	4.5 ± 0.4
+2	–17.9	+2.6	+8.1	41 ± 2	5.0	4.6 ± 0.4
+5	+11.9	+10.9	+11.6	51 ± 2	6.2	4.7 ± 0.3
+25	+26.8	+30.3	+31.4	54 ± 4	4.7	4.6 ± 0.3
	Average deviation from expected value					
Average values	6.2 ± 7.3	3.4 ± 2.8	5.5 ± 2.4	46 ± 3	5.7 ± 0.3	4.5 ± 0.4

**Figure 5:**
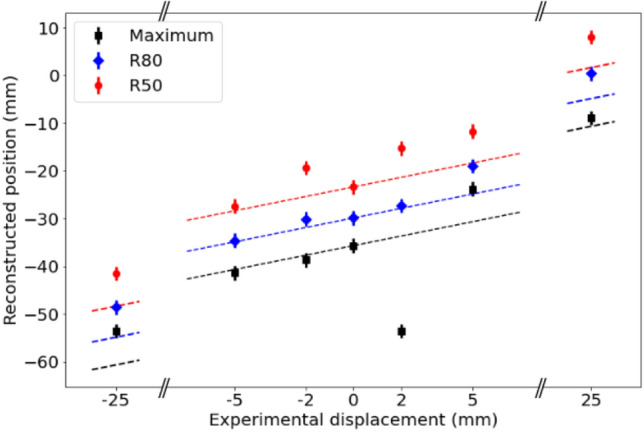
Range estimation for the six measured target positions using the maximum peak position (black), R80 (blue) and R50 (red) distances. Positions obtained from the longitudinal profiles at 4.4 MeV shown in Fig. 4. The error bars represent one voxel width. Dotted lines indicate the expected experimental positions, taking the values obtained from position 0 as a reference. The *x* axis is discontinued to include the two extreme points.

For a better visualization, the reconstructed maximum, R80 and R50 positions from the different measurements are plotted in Fig. 5. In this figure, discontinuous lines with slope 1 and containing the reference values of the maximum, R80 and R50 at position 0 are also drawn to indicate the expected reconstruction positions. The biggest deviation from the expected value is found in the reconstructed peak position from the measurement with a target displacement of +2 mm. As seen from its corresponding profile in Fig. 4b, in this case the reconstructed region with highest activity is distributed over an elongated area before the beam range, and the peak position is found at a longer distance from R80 and R50. As shown in Fig. 5, the reconstructed positions of R80 and R50 are in better agreement with the expected values, which indicates that these parameters might be more robust to statistical fluctuations for the determination of range shifts.

## Discussion

The aim of this work was to test the ability of our two-layer Compton camera to detect experimentally and quantify range shifts of proton beams. The results presented in the previous section show that this can be achieved by the current version of the prototype.

With the aim of recovering PG emission images from the experimental measurements, two aspects regarding the nature of the detected data have been addressed: the high background of coincidences produced by unwanted events and the spectral distribution of the PG that convey the sought signal information. Background reduction in the dataset before image reconstruction was accomplished through the implementation of a NN for event selection or rejection. After event selection, image reconstruction was performed using the four-dimensional (space and energy) model described in (Muñoz et al. 2020)^32^.

The NN was trained with synthetic data obtained from simulations. Results with simulated data show that the percentage of true signal increased from 7.6 to 10.3%, with a recall of 84.0%, after event selection with the NN. In relative terms, the ratio of true signal events is increased by 35.4%. In the reconstructed four-dimensional images (Fig. 1), this translates into a better recovery of the 4.4 MeV spectral line. From the spatial images and profiles obtained at this energy, it can be seen that the peak position is better determined after event selection with the NN. All the parameters studied to analyse the reconstructed images show an improvement when the dataset of events selected by the NN is employed (Table 2): the images obtained present a narrower lateral spread, more accurate positioning of the peak in both spatial and spectral dimensions, a lower background component and a higher CNR. When all events are included in the reconstruction, the reconstructed spectrum shows a peak of intensity at 4.8 ± 1.2 MeV. In this dataset, the 4.44 MeV peak is contaminated by the non-signal events generated by PG undergoing a e$^+$e$^-$ pair production followed by the detection of an annihilation photon. These background events cannot be properly reconstructed by the spectral algorithm and hinder the determination of the correct energy of emission. The peak at 4.44 MeV is better determined in the spectra reconstructed from the set of selected events, showing that the NN effectively reduces this kind of background. Given the enhancement produced by the used event selection technique, the use of different NN architectures with increasing complexity is also foreseen to study a possible enhancement of the method. Another studied technique for background reduction is the employment of time of flight (ToF) measurements to reject neutron contributions^49^. Since the timing performance of the current prototype does not allow using ToF information, the development of an experimental prototype with improved time resolution is under investigation.

After application of the trained classification NN to experimental data, the production distribution of PG has been reconstructed. The reconstructed four-dimensional image for the measurement at position 0 (Fig. 2), with higher statistics (13.9 $\cdot 10^4$ events after NN selection), can be analysed in more details. The recovered experimental spectrum is similar to the one obtained from simulations after event selection, with intensity peaks at 2.2 and 4.4 MeV.

In order to consider the contribution of all emission energies, an image of the emission distribution of all PGs can be obtained by integrating over the whole considered energy range (0.8–9.9 MeV, see the image reconstruction subsection). In the integrated image obtained from our measured data (Fig. 2a), a region of higher activity is recovered at the end of the beam range. Since the irradiated target is made of PMMA, with a high $^{12}$C component, the most intense PG line correlated to the beam range is emitted at 4.44 MeV, and this energy is the best suited for imaging in this case. In the image obtained at 4.4 MeV (Fig. 2b), an emission distribution along the beam path ending in a peak is reconstructed, beyond which the intensity quickly drops. Other energies, such as the emission of 6.13 MeV photons by $^{16}$O, are also expected to produce images correlated to the Bragg peak position in the irradiation of human tissue^50,51^. In the case of beam irradiations on targets of different material compositions, for which the 6.13 MeV line may become more important, the procedure followed in this work is expected to yield similar results as long as the NN for event selection is re-trained with an appropriately generated set of data.

For the detection of range shifts, data from the target at six different positions with respect to the system were taken. The PG emission distribution at the energy of 4.4 MeV was reconstructed in all cases. The longitudinal profiles extracted from the images show that the reconstructed distributions for the different positions are displaced according to the experimental shifts, demonstrating the system capability of detecting range shifts of at least 3 mm. Due to statistical fluctuations, in some cases the reconstructed longitudinal profiles present several peaks of high intensity before the distal edge. For this reason, the maximum peak position is not always the best indicator to detect range shifts. In the case of measurement at position P0+2 mm, a more extended region of high intensity is reconstructed at the end of the beam range, and the maximum of intensity is placed further from the distal edge than in the other measurements. The parameters R80 and R50 are also evaluated at the different positions. Based on the results obtained in this work, with the limited available statistics, the parameters R80 and R50 appear as more robust indicators of the beam range inside the target. As seen from Fig. 5, the calculated R80 and R50 values for the different measurements are shifted in the correct direction of the experimental target displacements in all cases. The best results are achieved using the R80 parameter, with an average relative deviation from the expected values of 3.4 mm.

The correct identification of 3 mm range shifts is compatible with the results reported in the literature with similar systems. In^52^, a shift of 2 mm is identified using a four-stage Compton camera based on CZT detectors. In that work, the first plane of the camera was placed at 15 cm from the beam, and the D2C filter^40^, based on the prior spatial knowledge of the beam position, is employed for event selection and assignment of the initial energy. Systems employing PG imaging modalities other than Compton cameras for beam range estimation, and more advanced towards their clinical application, have demonstrated higher precision at clinical rates with full-scale prototypes. In Xie et al.^21^, the authors claim that a precision of 2 mm can be achieved with a system based on knife-edge slit collimation using spot aggregation. In the case of Hueso-Gonzalez et al.^53^, a system for beam range monitoring using PG spectroscopy and a single-slab collimator is presented, and a precision of 1.1 mm at a 95% confidence level is reported using spot aggregation as well. While the results obtained with our current prototype still lie behind the ones obtained with more advanced systems, they show its viability to detect range shifts with the technology employed and its potential for performance improvement.

The results obtained demonstrate the feasibility of the employed technique to detect range shifts inside the target. While the technique is promising, the measurements reported in this work were performed with a total of 2.1 $\cdot 10^{11}$ protons, which is between 3 and 4 orders of magnitude more than the number used per spot in a clinical scenario. In order to reach the statistics achieved in the reported measurements in a clinical treatment scenario, the detection efficiency of the experimental prototype must be upgraded. New detectors with a detection area four times larger than the ones employed in this work are already under development. Using the methodology for the calculation of the system sensitivity validated in a previous work^54^, the efficiency of a two-plane Compton camera at 4.4 MeV with the new detectors is estimated to increase by a factor of around 14 with respect to the current prototype. The use of detectors with larger area will lead to an increase of the count rate. However, the number of fortuitous coincidences is also expected to be amplified, which will require an improvement of the event selection technique. To this end, NNs with more complex architectures will be investigated. In order to cope with higher measurement rate, new acquisition electronics are under investigation to be able to process the data. An additional factor of 2 can be gained when all two- and three-coincidence events can be measured^43^ and the detection efficiency can also be enhanced by reducing the distance between the beam and the first working plane. Even with the foreseen geometry optimization, spot aggregation might be required in order to reach the necessary statistics for range determination. In addition to the efficiency improvement, the larger detection surface, an optimized geometry and the use of all the available coincidences in a three-plane system is also expected to enhance the quality of the reconstructed images, improving the system capability of recovering the Bragg peak position and achieving similar results with fewer statistics. All these aspects are being addressed by the group for clinical applicability of a future prototype.

## Methods

### Experimental set-up

The Compton camera consists of three independent detectors operating in time coincidence. Each of these detectors is composed of a monolithic LaBr$_3$ scintillator crystal coupled to an array of 64 SiPMs. Crystals in the first two planes have an active volume of 25.8$\times$25.8$\times$5.0 mm$^3$, whereas the size of the third one is 36.0$\times$32.4$\times$10.0 mm$^3$. The coincidence time window used to associate events in different planes is 50 ns. A detailed description of the system can be found in (Barrio et al. 2017)^46^.

**Figure 6:**
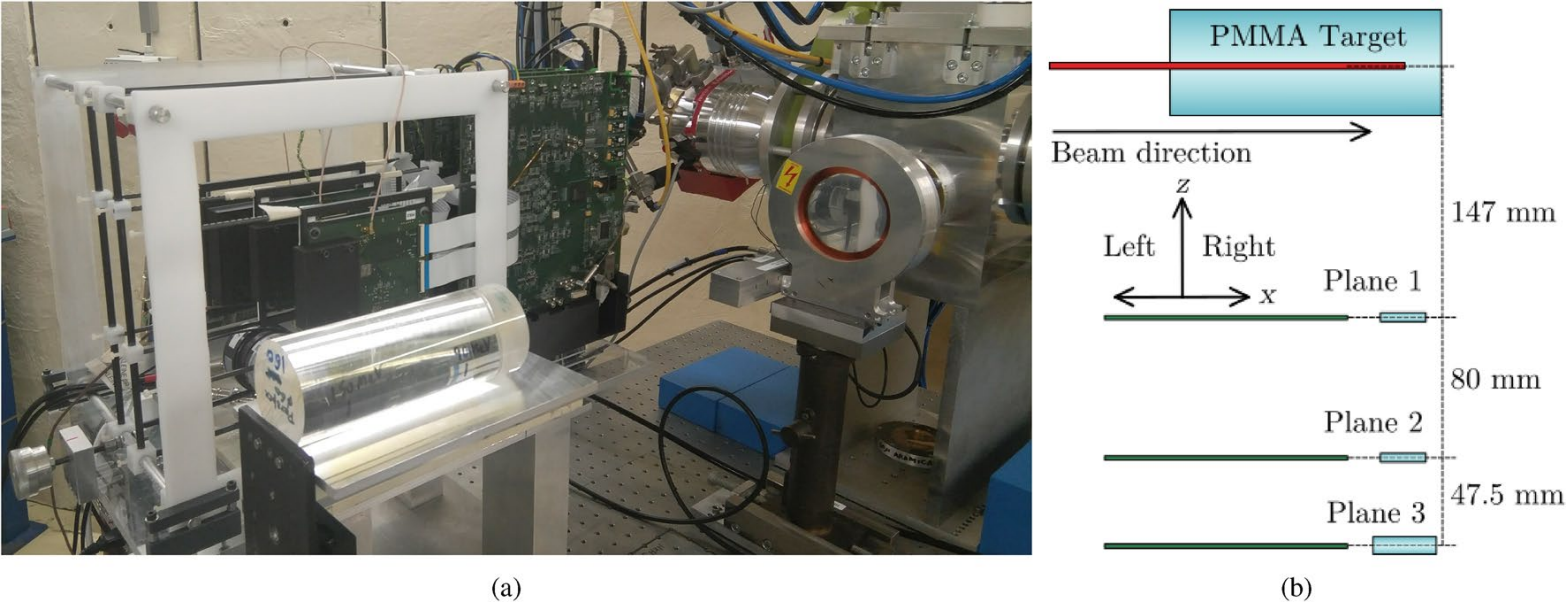
(**a**) Picture of the experimental set-up showing beam exit, PMMA target and Compton camera. (**b**) Diagram of relative distances in the experimental set-up.

Tests were performed on-beam at the irradiation facility of the AGOR cyclotron at KVI-Center for Advanced Radiation Technology, University of Groningen, the Netherlands. The AGOR cyclotron can deliver proton beams with energies between 20 MeV and 190 MeV, with a radio frequency of 55 MHz. The experiments in this work were performed with proton beams with fixed energy of 150 MeV and an irradiation rate of about 10$^8$ protons s$^{-1}$, monitored by an open air ionization chamber that allows measuring the beam intensity during irradiation. At this energy, the Bragg peak position is calculated at 133.5 mm from the entrance surface in the PMMA target. The target was placed on a motorized stage using a Newport LTA-HS high speed motorized actuator, with an accuracy of ±5 $\mu$m, which allows its displacement over a range of 50 mm. The collimated beam has an estimated spot size of 7 mm FWHM in both horizontal and vertical directions at the target entrance. Changes in the beam range due to energy losses in air are estimated below 50 $\mu m$ for the target displacements measured in this study, and can therefore be neglected. The beam broadening in air for the distance between the two extreme target positions is calculated to be 0.38 mm FWHM, which is not expected to cause significant differences in the reconstructed beam ranges. The employed beam intensity was selected due to the limited count rate of the current version of the experimental prototype.

A picture of the experimental set-up is shown in Fig. 6a, where the beam exit, PMMA target and Compton camera are visible. A diagram of the system positioning with respect to the target and particle beam can be seen in Fig. 6b. Although the system was assembled with three planes for the entire experiment, only two-detector coincidence events from the pair formed by the second and third detectors have been used for image reconstruction, due to the worse performance of the first detector observed in the data analysis.

### Simulations

The experiment was simulated with GATE^48^ version 8.2, using Geant4^55^ version 10.05, to gain insight into the measured data. A 150 MeV proton beam impinging on a PMMA cylinder with the same dimensions as the experimental target was simulated. The physical processes and probabilities were defined using the predefined *QGSP-BIC-HP-EMZ* physics list, which yielded results compatible with the experimental measurements. For these simulations, the different detector planes were placed at the distances corresponding to the experimental measurements. In order to increase the statistics and reduce simulation time for this study, the area of the three planes was set to 100 $\times$ 100 mm$^2$. For this study, a total of $4 \cdot 10^{10}$ protons have been simulated, distributed among 2000 different simulations. Each simulation was performed with a total of $2 \cdot 10^{7}$ primary protons, which took 35 hours of computing on a single core. The total computing time needed to achieve the obtained statistics was approximately $7 \cdot 10^{4}$ hours, computed in parallel on 20 different cores on the GRID computing farm at IFIC^56^. The precision achieved by the NN as a function of the number of events used for training reached a plateau after 80% of the simulated events, so the results are not expected to improve significantly for higher statistics with the employed NN architecture.

The purpose of the simulations is twofold: in the first place, to use the realistic simulated spectra, including all by-products generated during irradiation, to calibrate the energy response of our system in the measured data; in the second place, to use the energy distributions of signal and background events to train the NN designed for event selection to maximize the ratio of true signal events in the data employed for image reconstruction. For the purpose of event selection, the simulated data are first classified as true signal or background. The set of true signal events includes all events in which a photon generated during irradiation reaches the system and undergoes a Compton scatter in the scatterer, followed by any interaction in the absorber. All other events, including photon interactions with wrong ordering and those produced by other particles, are classified as background. It should be noted that this criterion classifies as signal all events produced by any photon reaching the system, irrespective of its creation process, so secondary photons are also classified as signal. In order to exclude low energy photons produced by radiative processes, which are not correlated to the deposited dose^57^, a low energy threshold of 750 keV was applied in the combined deposited energy before event selection.

### Calibration of measured spectra

**Figure 7:**
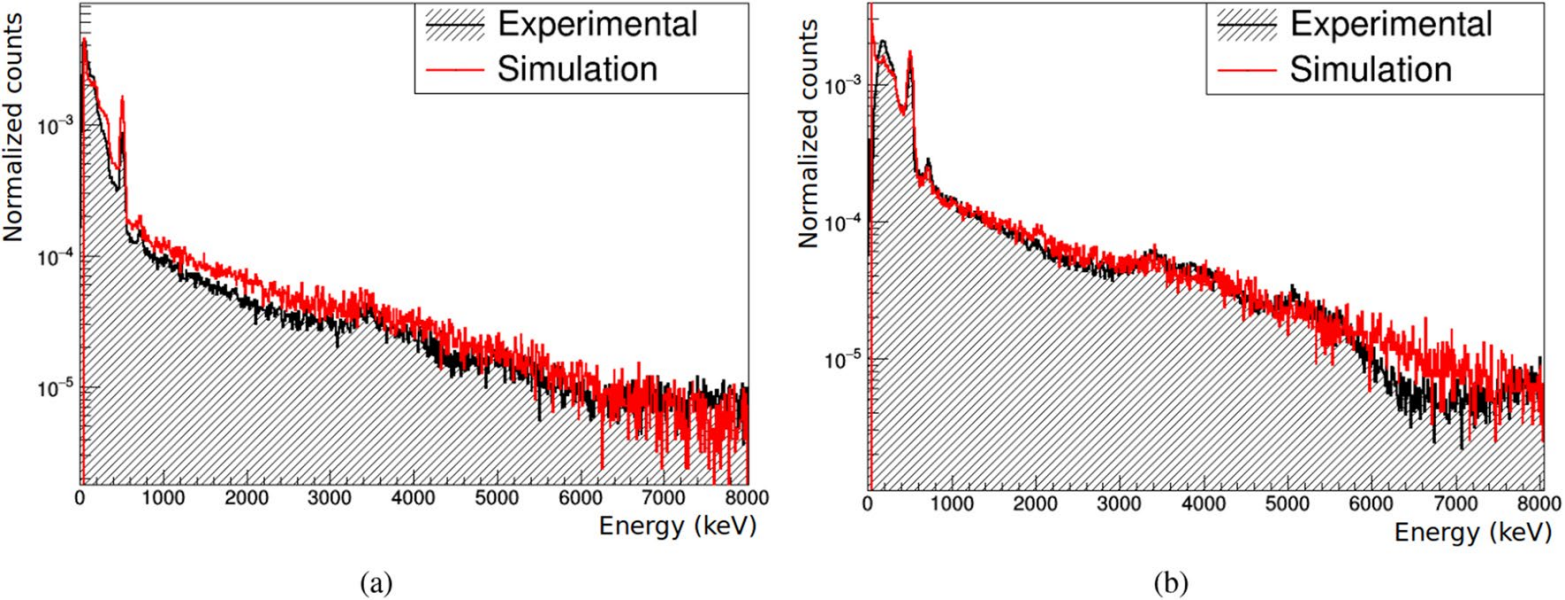
Experimental and simulated spectra in *singles* mode measured in the second (**a**) and third (**b**) planes.

The individual detector planes have been calibrated using the spectra measured in-beam in *singles* mode, that is, working independently from the other planes (not in time coincidence). The measured energy response of each detector is calibrated by comparing the identifiable peaks (511, 718 and 3417 keV) to those obtained in simulations. The calibrated spectra are plotted in Fig. 7, showing good agreement with simulated results. This calibration has been maintained for the measurements in time coincidence, from which images of the PG emission distribution have been reconstructed.

### Event selection

A NN has been implemented using TensorFlow^58^ to the end of reducing background events. The energy measured in the two individual planes is fed to the NN as input, and its final output is a binary classificator to either accept or reject the event. The NN contains two fully connected hidden layers of 80 and 40 nodes respectively, employing the ReLU (Rectified Linear Unit) activation function. A dropout layer with a rate of 25% after the first layer is included for regularization in the training stage. The output value of the NN is obtained after a sigmoid activation in the output layer, which yields a numeric result between 0 and 1. The NN prediction for an event is 1 (true signal) if its output value is greater than 0.5 and 0 (background) otherwise. The NN is trained by minimizing the binary cross entropy loss function between actual and predicted event labels.

The training and validation sets used to optimize the NN parameters were obtained from simulations and labeled as signal or background as described in the simulations subsection. After training, the NN was employed for event selection in the experimentally measured datasets prior to image reconstruction.

### Image reconstruction

Images have been reconstructed by means of a spectral reconstruction method that estimates the spatial and energy distributions of the measured photons. The method considers a four-dimensional (three spatial coordinates and energy) field of view (FoV), in which the four-dimensional voxels are associated to an emission energy. Each measured event is backprojected to the FoV using all the possible emission energies in the range considered. Taking into account the Compton scattering formula, this means that every event generates a set of conical surfaces, each corresponding to a single emission energy. The different conical surfaces are modelled as a dense set of rays and backprojected onto the FoV using a ray-tracing technique^59^. A detailed description of the implementation of the method and calculation of the system and sensitivity matrices can be found in (Muñoz et al. 2020)^32^.

Spatial-spectral images are calculated with the standard list-mode Maximum Likelihood Expectation Maximization (MLEM) algorithm. In this work, all images have been reconstructed using a FoV with $101 \times 101 \times 1$ spatial voxels of $3 \times 3 \times 3$ mm$^3$ and 92 energy bins of 0.1 MeV, ranging from 0.8 to 9.9 MeV. A median filter with size $3 \times 3 \times 1 \times 3$ (3 voxels in the *x*,*y* spatial dimensions and 3 voxels in the energy dimension) and constant weights is applied to the current image estimate between successive iterations. All reconstructed images shown in this work correspond to iteration 50 of the algorithm, after convergence is reached.

The reconstructed four-dimensional images provide information on both the spatial distribution and the spectral emission of the measured dataset. If the spectral domain of the image is integrated out, the spatial distribution for the whole energy range is obtained. Conversely, the reconstructed energy spectrum can be recovered by integrating out the spatial extent of the image. In addition, the spatial distribution reconstructed for a particular emission energy can be extracted by selecting the corresponding three-dimensional slice. Since 4.439 MeV is the most prominent PG emission energy correlated to the absorbed dose, in this work the spatial image obtained for the energies between 4.3 and 4.5 MeV has been used for the estimation of range shifts within the irradiated target.

### Image assessment and range estimation

A method for range estimation similar to the one employed in Ortega et al.^41^ has been followed to calculate the distal fall-off. Longitudinal profiles along the beam direction are generated by integrating the reconstructed voxels in a 3 cm wide slice in the *y* direction (perpendicular to the beam). This width is chosen to include the activity of the beam lateral spread. From these longitudinal profiles, in order to compare the gradient of the curve, different depths have been considered: the depth at the maximum peak and the depths at 80% (R80) and 50% (R50) height of the maximum peak. Given that images are reconstructed with a fixed number of voxels, the minimum resolution of the profiles is limited to the voxel size. For the calculation of the range estimation parameters, the peak position is taken as the center of the corresponding voxel, and R80 and R50 are calculated from a linear interpolation between the voxels immediately above and below the heights of 80 and 50 percent of the peak.

In addition to range estimations, other figures of merit are used to quantify the reconstructed images. The beam lateral spread is calculated as the FWHM of the beam transverse profiles, obtained at the maximum position with a width of 10 voxels in the *x* direction. The contrast-to-noise ratio (CNR) of the images is calculated as $CNR = \vert S - \mu \vert / \sigma$, where *S* is the intensity at the peak position and $\mu$ and $\sigma$ are respectively the mean value and standard deviation of the background. For this calculation, the background values are taken from the voxels located outside a region of 150 mm along the beam direction and 60 mm in its perpendicular direction to exclude the lateral spread of the reconstructed activity.

## Conclusion

The tests reported in this work were aimed at assessing the performance of MACACO II with experimental beam data, using a spectral method to estimate the PG energy and a NN for event selection and background reduction. The results obtained from in-beam measurements demonstrate that the system is able to detect range shifts in the irradiated target. Images of the emission distribution of 4.4 MeV prompt gammas have been recovered, allowing calculation of the distal fall-off and identification of target displacements of 3 mm.
