# Author Correction: Proton range verification with MACACO II Compton camera enhanced by a neural network for event selection

**DOI:** 10.1038/s41598-021-03252-5

**Published:** 2021-12-07

**Authors:** Enrique Muñoz, Ana Ros, Marina Borja-Lloret, John Barrio, Peter Dendooven, Josep F. Oliver, Ikechi Ozoemelam, Jorge Roser, Gabriela Llosá

**Affiliations:** 1 Instituto de Física Corpuscular (IFIC), CSIC/Universitat de València, València, Spain; 2 KVI-Center for Advanced Radiation Technology, University of Groningen, Groningen, The Netherlands; 3 Department of Radiation Oncology, University Medical Center Groningen, University of Groningen, Groningen, Netherlands

Correction to: *Scientific Reports* 10.1038/s41598-021-88812-5, published online 29 April 2021

The original version of this Article contained an error in Reference 46, which was incorrectly given as:

Barrio, J. et al. Performance improvement tests of MACACO: a Compton telescope based on continuous crystals and SiPMs. Nuclear Instruments and Methods in Physics Research Section A: Accelerators, Spectrometers, Detectors and Associated Equipment (2017).

The correct reference is listed below:

Barrio, J. *et al*. Performance improvement tests of MACACO: A Compton telescope based on continuous crystals and SiPMs. *Nuclear Instruments and Methods in Physics Research Section A: Accelerators, Spectrometers, Detectors and Associated Equipment.* **912,** 48–52. https://doi.org/10.1016/j.nima.2017.10.033 (2018).

The original Article has been corrected.

